# Analysis of Stress Concentration in Functionally Graded Plates with Linearly Increasing Young’s Modulus

**DOI:** 10.3390/ma16216882

**Published:** 2023-10-26

**Authors:** Hassan Mohamed Abdelalim Abdalla, Daniele Casagrande, Francesco De Bona

**Affiliations:** Polytechnic Department of Engineering and Architecture, University of Udine, Via delle Scienze 206, 33100 Udine, Italy; daniele.casagrande@uniud.it (D.C.); francesco.debona@uniud.it (F.D.B.)

**Keywords:** functionally graded plates, circular hole, stress concentration, linear Young’s modulus, stress analysis

## Abstract

In this article, the strain and stress analyses of functionally graded plates with circular holes that are subject to a uniaxial far-field traction load are analytically considered. The Young’s modulus is assumed to vary linearly along the radial direction around the hole. The adoption of such a type of inhomogeneity variation can be justified as follows. Firstly, and among all the possible variations of stiffness, the linear one is indeed the simplest inhomogeneity distribution. Surprisingly however, according to our knowledge extent, the associated elastic fields were not yet addressed in the literature. Secondly, a linearly varying stiffness could reasonably imply a remarkable advantage from a technological point of view. In fact, unlike nonlinearly varying stiffness plates, manufacturing routes are only required to handle constant variations throughout the radial domain. After recalling the basic equations for plane stress elasticity, the displacement, strain, and stress fields around the hole were numerically tackled and discussed for different stiffness ratios. A comparison was also carried out with other Young’s modulus distributions that have been commonly employed in the literature.

## 1. Introduction

The stress evaluation in plates endowed with geometrical discontinuities constitutes one of the major problems in mechanical engineering. Kirsch [1] analytically assessed the stress concentration factor at the rim of a circular hole under the assumption of a homogeneous, linearly elastic and isotropic material under a far-field, in-plane traction load. He found that the hoop stress, at the rim of the hole, comes out to be identically three times the applied load when the dimension of the hole is very small compared to the dimensions of the plate. Since then, the interest in reducing such a factor by abandoning the above assumptions has been considerably increasing. Analytical and numerical approaches have yielded results for both infinite and finite plates. For instance, an overview of the analytic methods describing the elastic stresses in two-dimensional flat anisotropic plates with notches is presented in [2]. In [3], the authors report studies on the stress concentration arising near to holes and different cutouts when the panels are made of fiber composite materials. The authors in [4] derived closed-form solutions for circularly notched composite laminates that were subject to tensile loading. The mechanical behavior of composite finite laminates endowed with open holes was assessed by analytical methods in [5]. In [6], the authors analyzed the stress concentration at the edge of a circular hole in sheets with different widths by means of the finite element method.

On the other hand, the increasing interest in the so-called functionally graded materials has been gaining considerable interest by researchers, since the adoption of such a material concept could lead to remarkable stress and strain enhancements in several mechanical models. For instance, the vibrations of thin, microstructured, functionally graded plates were studied in [7,8] by means of the so-called tolerance modeling method. In particular, free vibration frequencies bands were analyzed, and the effect of various distribution functions on the plate properties was shown. In [9], the thermomechanical stresses occurring in functionally graded rotating hollow disks were analytically computed and numerically optimized for a particular gradation law along the radial direction of the disk. Lightweight and economical designs for functionally graded rotating disks of a fixed mass based on the limitation of their angular speeds before material yielding can be found in [10]. The mass values, strains, and stresses of rotating disks subject to thermal loads were numerically enhanced within a multiobjective optimization study in [11]. In [12], the thermal and mechanical stresses occurring in functionally graded pressurized cylindrical vessels with spherical domes were computed and optimized using a commercial finite-element-based software. The flexural, free vibration, and buckling responses of two-dimensional functionally graded curved beams whose material properties in both directions were varied according to power law distributions were numerically studied in [13]. In [14], the maximization of the sound transmission loss across a functionally graded material in the form of a cylindrical shell was conducted using a genetic algorithm and compared to the responses associated with several traditional materials.

As far as plates with geometrical discontinuities (e.g., a circular hole) are concerned, the literature offers substantial works where the stress analysis has been carried out either analytically or numerically—once the homogeneity assumption is dropped. For instance, the effect of the material inhomogeneity on the stress field due to circular holes was studied in [15] by means of the finite element method. In particular, a considerable reduction in the stress concentration factor was obtained by allowing the Young’s modulus to vary in a power law or exponential fashion. In [16], the stress concentration of an infinite plate subjected to uniform biaxial tension and pure shear was analytically obtained by considering an exponential variation of Young’s modulus throughout the radius. In [17], closed-form solutions for the stress fields of functionally graded plates subject to uniform far-field tensile traction were derived. The elastic response of a functionally graded annular ring inserted around the hole of a homogeneous plate was derived analytically in [18,19] under different far-field loading conditions. The stress concentration factor measured for exponentially graded plates with circular holes and subject to biaxial loads was studied in [20] by means of the boundary element method. The main results of these works can be summarized by the following two statements:

- The stress concentration factor decreases only when the stiffness progressively increases away from the circular hole;
- The effect of the variation in the Poisson’s ratio on the stress distribution is negligible.

These two findings entail a remarkable rule of thumb for designers and stress analysts.

Despite the enhanced stress results arising from the relaxation of the homogeneity assumption, it is surprising that studies on the stress concentration behavior in functionally graded plates with a circular hole whose Young’s modulus linearly increases are not considered in the literature. Although a number of works that adopted a linear (or piecewise linear) stiffness distribution can be found in the literature for different mechanical models, e.g., [21,22,23,24], such a distribution has been so far neglected as far as stress concentration problems are concerned. Supposedly, a linearly increasing stiffness is the simplest variation form among all of the possible variations and could present an advantage from a manufacturing viewpoint with respect to its nonlinear counterparts. According to [25], this could be one of the desired characteristics when manufacturing such materials, since steep variations in the stiffness along the radial domain could be, reasonably, more costly and more difficult to obtain.

The article is organized as follows. Section 2 recalls the governing equations for the elastic behavior of the functionally graded plate. A boundary value problem is obtained and solved numerically in Section 3. The numerical solutions for the displacement, strain, and stress fields are presented in graphical form and discussed in Section 4. Finally, conclusions are drawn in Section 5.

## 2. Governing Equations

**Figure 1:**
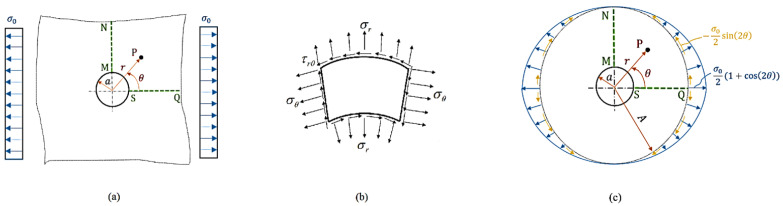
(**a**) A schematic representation of an infinite plate with a circular hole of radius *a* subject to a far-field uniaxial traction load $\sigma_{0}$. (**b**) An infinitesimal element along the line MN demonstrating the directions of plane stresses. (**c**) The transcription of the boundary conditions and the definition of the limit radius *A*.

Consider a locally isotropic, linearly elastic, and functionally graded infinite plate with a circular hole of radius *a* and that is subject to a far-field uniaxial load $\sigma_{0}$, as shown in Figure 1a, where the generic point P is described by the polar coordinate system (r,θ), whose origin is at the center of the circular hole.

Let SQ and MN denote the horizontal and vertical lines, respectively, associated with the polar angles θ=0 and θ=π/2, respectively. Moreover, let the inhomogeneity be described by a radially linear variation in the Young’s modulus, of slope *e*, which is given by

(1) $$E\left(r\right)=E_{a}+e\left(r-a\right)\;,$$

where $E_{a}$ denotes the Young’s modulus at r=a.

By letting the thickness of the plate be sufficiently small to the point that the stress state results are two-dimensional (plane stress condition) and considering no body forces, the equilibrium equations in terms of the plane stresses in the polar coordinates read as [26]

(2) $$\left\{\begin{array}{c} \frac{\partial \sigma_{r}\left(r,\theta \right)}{\partial r}+\frac{1}{r}\frac{\partial \tau_{r\theta }\left(r,\theta \right)}{\partial \theta }+\frac{\sigma_{r}\left(r,\theta \right)-\sigma_{\theta }\left(r,\theta \right)}{r}=0\;,\\ \frac{\partial \tau_{r\theta }\left(r,\theta \right)}{\partial r}+\frac{1}{r}\frac{\partial \sigma_{\theta }\left(r,\theta \right)}{\partial \theta }+\frac{2}{r}\tau_{r\theta }\left(r,\theta \right)=0\;, \end{array}\right.$$

where $\sigma_{r}$, $\sigma_{\theta }$, and $\tau_{r\theta }$ are, respectively, the radial, hoop, and shear stresses, whose orientations are shown in Figure 1b. These elastic stresses are, in turn, linked to the corresponding strains by the plane stress constitutive relations, namely [26],

(3) $$\left\{\begin{array}{c} \sigma_{r}\left(r,\theta \right)=\frac{E(r)}{1-\nu^{2}}\left(\varepsilon_{r}\left(r,\theta \right)+\nu \varepsilon_{\theta }\left(r,\theta \right)\right)\;,\\ \sigma_{\theta }\left(r,\theta \right)=\frac{E(r)}{1-\nu^{2}}\left(\varepsilon_{\theta }\left(r,\theta \right)+\nu \varepsilon_{r}\left(r,\theta \right)\right)\;,\\ \tau_{r\theta }\left(r,\theta \right)=\frac{E(r)}{2(1+\nu )}\gamma_{r\theta }\left(r,\theta \right)\;, \end{array}\right.$$

where ν is Poisson’s ratio (which is supposed to be constant for simplicity) and $\varepsilon_{r}$, $\varepsilon_{\theta }$, and $\gamma_{r\theta }$ are the radial, hoop, and shear strains, respectively. From Figure 1a, the boundary conditions can be described as follows:

1. At the hole periphery, both the radial and shear stresses are zero for all values of θ;
2. At a sufficiently large distance from the hole, the radial and shear stresses on the MN approach zero;
3. At a sufficiently large distance from the hole, the radial and shear stresses on the SQ tend to $\sigma_{0}$ and to zero, respectively.

Furthermore, strains are linked to the plane displacement field through the following kinematic relations in polar coordinates [26]:

(4) $$\left\{\begin{array}{c} \varepsilon_{r}\left(r,\theta \right)=\frac{\partial u(r,\theta )}{\partial r}\;,\\ \varepsilon_{\theta }\left(r,\theta \right)=\frac{u(r,\theta )}{r}+\frac{1}{r}\frac{\partial v(r,\theta )}{\partial \theta }\;,\\ \gamma_{r\theta }\left(r,\theta \right)=\frac{1}{r}\frac{\partial u(r,\theta )}{\partial \theta }+\frac{\partial v(r,\theta )}{\partial r}-\frac{v(r,\theta )}{r}\;, \end{array}\right.$$

where *u* and *v* are the radial and hoop components of the displacement field, respectively. It is worthwhile to mention that these displacement functions have to be sagaciously chosen so that the elastic strains obey the following compatibility equation [26]:

(5) $$\frac{\partial^{2}\varepsilon_{\theta }\left(r,\theta \right)}{\partial r^{2}}+\frac{1}{r^{2}}\frac{\partial^{2}\varepsilon_{r}\left(r,\theta \right)}{\partial \theta^{2}}+\frac{2}{r}\frac{\partial \varepsilon_{\theta }\left(r,\theta \right)}{\partial r}-\frac{1}{r}\frac{\partial \varepsilon_{r}\left(r,\theta \right)}{\partial r}=\frac{1}{r}\frac{\partial^{2}\gamma_{r\theta }\left(r,\theta \right)}{\partial r\partial \theta }+\frac{1}{r^{2}}\frac{\partial \gamma_{r\theta }\left(r,\theta \right)}{\partial \theta }\;.$$

If the Young’s modulus is assumed to be constant in Equations (1)–(4) (namely, homogeneous plates), the elastic stresses can be determined analytically. More precisely, by splitting the elastic problem into a biaxial (axisymmetric) problem and a pure shear problem, the superposition principle and the introduction of the Airy stress function lead to the well-known Kirsch stress field [26]:

(6) $$\left\{\begin{array}{c} \sigma_{r}\left(r,\theta \right)=\frac{\sigma_{0}}{2}\left(1-\frac{a^{2}}{r^{2}}\right)+\frac{\sigma_{0}}{2}\left(1-\frac{4a^{2}}{r^{2}}+\frac{3a^{4}}{r^{4}}\right)\cos2\theta \;,\\ \sigma_{\theta }\left(r,\theta \right)=\frac{\sigma_{0}}{2}\left(1+\frac{a^{2}}{r^{2}}\right)-\frac{\sigma_{0}}{2}\left(1+\frac{3a^{4}}{r^{4}}\right)\cos2\theta \;,\\ \tau_{r\theta }\left(r,\theta \right)=-\frac{\sigma_{0}}{2}\left(1+\frac{2a^{2}}{r^{2}}-\frac{3a^{4}}{r^{4}}\right)\sin2\theta \;. \end{array}\right.$$

Equation (6) forms one of the few closed-form solutions in the theory of elasticity, whose expressions are twofold. Firstly, it demonstrates that if *r* and θ tend to *a* and π/2, respectively, the stress concentration factor, namely, the ratio between the hoop stress and the applied load, is identically three. Another important consequence consists of the derivation of the expression for the far-field radial and shear stresses at a sufficiently large distance from the hole by making *r* tend to +∞, namely,

(7) $$\left\{\begin{array}{c} \lim_{r\to +\infty }\sigma_{r}\left(r,\theta \right)=\frac{\sigma_{0}}{2}\left(1+\cos2\theta \right)\;,\\ \lim_{r\to +\infty }\tau_{r\theta }\left(r,\theta \right)=-\frac{\sigma_{0}}{2}\sin2\theta \;. \end{array}\right.$$

Equation (7) not only transcribes the boundary conditions as a function of θ, but also allows for a transformation of the rectangular domain into a circular one as long as a limit radius, hereinafter denoted by *A*, is provided (see Figure 1c). The latter can be defined by referring to a fixed threshold of the discrepancy of the stress concentration factor associated with finite and infinite homogeneous plates. For instance, in [27], the solution to the problem for radially inhomogeneous material was carried out with a limit radius that was ten times the radius of the hole. Such a value, for homogeneous plates, yields a discrepancy in the stress concentration factor of less than 3%. In the present study, we took as the limit radius the double of such a value.

Next, the analytical derivation for the elastic displacement field, and therefore the strains and stresses from Equations (3) and (4), is shown by adopting a set of compatible displacement functions, in the sense of Equation (5), which is borrowed from the generalized Michell solution in the polar coordinates [28]. Three coupled differential equations are obtained, whose analytical treatment is cumbersome, thus enforcing one to resort to a numerical tool when considering the resulting boundary value problem (BVP).

## 3. Solution of the Problem

Let the set of radial and hoop displacements be a function of *r* and θ, which is given by [27]:

(8) $$\left\{\begin{array}{c} u\left(r,\theta \right)=\bar{u}\left(r\right)+\hat{u}\left(r\right)\cos2\theta \;,\\ v\left(r,\theta \right)=\hat{v}\left(r\right)\sin2\theta \;. \end{array}\right.$$

It is easy to show that the radial displacements along the MN and SQ are given by $\bar{u}\left(r\right)-\hat{u}\left(r\right)$ and $\bar{u}\left(r\right)+\hat{u}\left(r\right)$, respectively. Moreover, it is required that $\bar{u}\left(A\right)-\hat{u}\left(A\right)$ be strictly negative, thus exhibiting the well-known Poisson’s effect. Based on the kinematic relations (4), the elastic strains read as follows:

(9) $$\left\{\begin{array}{c} \varepsilon_{r}={\bar{u}}^{\prime }+{\hat{u}}^{\prime }\cos2\theta \;,\\ \varepsilon_{\theta }=\frac{\bar{u}}{r}+\left(\frac{\hat{u}}{r}+2\frac{\hat{v}}{r}\right)\cos2\theta \;,\\ \gamma_{r\theta }=-\left(\frac{\hat{v}}{r}-{\hat{v}}^{\prime }+2\frac{\hat{u}}{r}\right)\sin2\theta \;, \end{array}\right.$$

where the dependence on *r* and θ are omitted for the sake of light notation, and a prime denotes a first derivative with respect to *r*. It is worth noting that Equation (9) satisfies the compatibility condition (5) identically, thus suggesting promising candidates for the description of the displacement field. Hence, the elastic stress field reads as follows:

(10) $$\left\{\begin{array}{c} \sigma_{r}=\frac{E}{1-\nu^{2}}\left[{\bar{u}}^{\prime }+\nu \frac{\bar{u}}{r}+\left({\hat{u}}^{\prime }+\nu \frac{\hat{u}}{r}+2\nu \frac{\hat{v}}{r}\right)\cos2\theta \right]\;,\\ \sigma_{\theta }=\frac{E}{1-\nu^{2}}\left[\frac{\bar{u}}{r}+\nu {\bar{u}}^{\prime }+\left(\frac{\hat{u}}{r}+2\frac{\hat{v}}{r}+\nu {\hat{u}}^{\prime }\right)\cos2\theta \right]\;,\\ \tau_{r\theta }=-\frac{E}{2(1+\nu )}\left(\frac{\hat{v}}{r}-{\hat{v}}^{\prime }+\frac{2\hat{u}}{r}\right)\sin2\theta \;, \end{array}\right.$$

whose substitution in the equilibrium relations of (2) yields the following three second-order linear differential and coupled equations:

(11) $$\left\{\begin{array}{c} {\hat{u}}^{\prime \prime }+\alpha_{1}{\hat{u}}^{\prime }+\alpha_{2}{\hat{v}}^{\prime }+\alpha_{3}\hat{u}+\alpha_{4}\hat{v}=0\;,\\ {\bar{u}}^{\prime \prime }+\alpha_{1}{\bar{u}}^{\prime }+\alpha_{6}\bar{u}=0\;,\\ {\hat{v}}^{\prime \prime }+\alpha_{1}{\hat{v}}^{\prime }+\alpha_{7}{\hat{u}}^{\prime }+\alpha_{5}\hat{v}+\alpha_{8}\hat{u}=0\;, \end{array}\right.$$

where $\alpha_{i}$ (i=1,2,…,8) are functions that depend on the distribution of the mechanical properties along the radial direction. Precisely, they are given by the following:

(12) $$\left\{\begin{array}{c} \alpha_{1}=\frac{rE^{\prime }+E}{rE}\;,\\ \alpha_{2}=\frac{\nu +1}{r}\;,\\ \alpha_{3}=-\frac{3-2\nu }{r^{2}}+\frac{\nu E^{\prime }}{rE}\;,\\ \alpha_{4}=-\frac{3-\nu }{r^{2}}+\frac{2\nu E^{\prime }}{rE}\;,\\ \alpha_{5}=-\frac{E^{\prime }}{rE}+\frac{9+\nu }{\left(\nu -1\right)r^{2}}\;,\\ \alpha_{6}=\frac{\nu E^{\prime }}{rE}-\frac{1}{r^{2}}\;,\\ \alpha_{7}=\frac{2\nu +2}{(\nu -1)r}\;,\\ \alpha_{8}=-\frac{2E^{\prime }}{rE}+\frac{6-2\nu }{\left(\nu -1\right)r^{2}} \end{array}\right.$$

Finally, as derived from Equation (10), the boundary conditions at the hole periphery, as well as at the limit radius, can be written in terms of $\bar{u}$, $\hat{u}$, and $\hat{v}$ as follows:

(13) $$\left\{\begin{array}{c} a{\bar{u}}^{\prime }\left(a\right)+\nu \bar{u}\left(a\right)=0\;,\\ a{\hat{u}}^{\prime }\left(a\right)+\nu \hat{u}\left(a\right)+2\nu \hat{v}\left(a\right)=0\;,\\ a\hat{v}\left(a\right)+a{\hat{v}}^{\prime }\left(a\right)-2\hat{u}\left(a\right)=0\;,\\ A{\bar{u}}^{\prime }\left(A\right)+\nu \bar{u}\left(A\right)-\frac{\sigma_{0}}{2}\frac{1-\nu^{2}}{E_{A}}=0\;,\\ A{\hat{u}}^{\prime }\left(A\right)+\nu \hat{u}\left(A\right)+2\nu \hat{v}\left(A\right)-\frac{\sigma_{0}}{2}\frac{1-\nu^{2}}{E_{A}}=0\;,\\ A\hat{v}\left(A\right)+A{\hat{v}}^{\prime }\left(A\right)-2\hat{u}\left(A\right)-\frac{\sigma_{0}\left(1+\nu \right)A}{E_{A}}=0\;, \end{array}\right.$$

where $E_{A}$ is the Young’s modulus at the limit radius, which is evaluated by substituting r=A in Equation (1).

The system of differential equations in Equation (11) and the boundary conditions (13) form a BVP, whose solution can be carried out numerically using a dedicated solver [29]. More precisely, the differential equations in Equation (11) are integrated on a mesh formed by *N* points, which divides the interval of integration into subintervals. A mesh convergence study led us to take N=200. The collocation polynomial approximating the solutions provides C^1^ continuous solutions. The solver determines a numerical solution by solving a global system of algebraic equations resulting from the boundary conditions (13), as well as the collocation conditions imposed on all the subintervals. The solver then estimates the error of the numerical solution on each subinterval; if the solution does not satisfy the tolerance criteria, the solver adapts the mesh and repeats the process.

## 4. Numerical Results

In this section, a numerical solution for the above mentioned BVP is presented and shown in graphical form. For simplicity, let the Young’s modulus linear distributions be described by the ratio $E_{A}/E_{a}$. Consider four instances of this ratio: One of them is associated with the value $E_{A}/E_{a}=1$ referring to the homogeneous case for the mere sake of comparison, while the others are 5, 10, and 20. Unless explicitly specified, let the dashed lines refer to numerical solutions associated with the homogeneous case. The derivation of the stresses in units of MPa requires one to express the stiffness in the same unit, the radius of the hole *a*, the limit radius *A* in mm, and the slope *e* in Equation (1), which is given by

(14) $$e=\frac{E_{A}-E_{a}}{A-a}\;,$$

in MPa/mm. However, displacements, strains, and stresses are hereinafter normalized with respect to $A\sigma_{0}/E_{a}$, $\sigma_{0}/E_{a}$, and $\sigma_{0}$, respectively.

**Figure 2:**
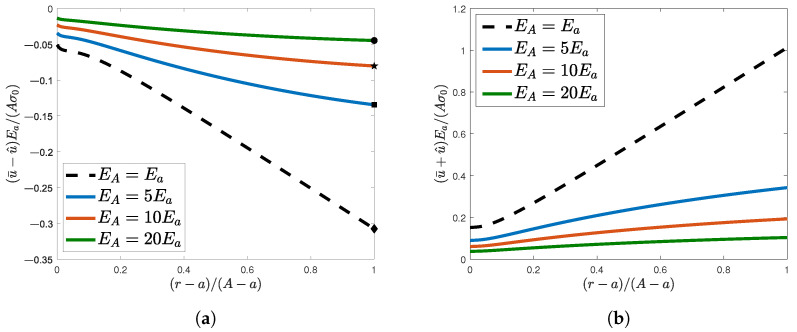
Normalized radial displacements along (**a**) MN and (**b**) SQ lines as $E_{A}/E_{a}$ increased. The Poisson’s effect is highlighted by scatter points in (**a**).

For clarity of exposition, the radial displacement fields along the MN and SQ are shown in Figure 2a,b respectively, which show the quantities $\bar{u}-\hat{u}$ and $\bar{u}+\hat{u}$, respectively. It is worth noting that the radial displacement along the MN was negative along the lines and up to the limit radius (whose values are highlighted by the scatter points in Figure 2a), thus confirming the Poisson’s effect when the plate is subject to a far-field traction load. As far as the displacement along the SQ at the limit radius is concerned, it is not difficult to notice that it decreased as long as the stiffness ratio $E_{A}/E_{a}$ reached higher values, which is in agreement with the physical idea of the corresponding continuously hardened plates.

**Figure 3:**
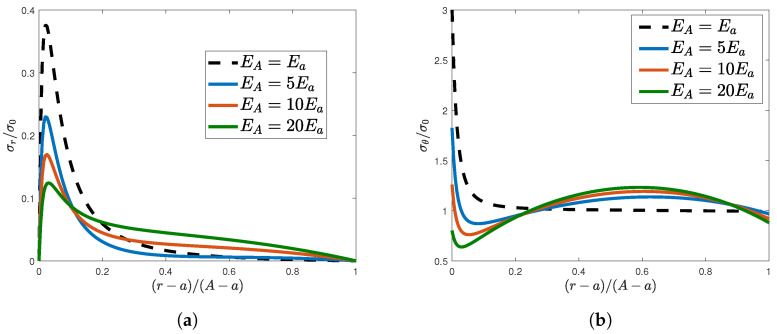
Normalized elastic stresses along the vertical MN line: (**a**) Radial and (**b**) hoop stresses.

Figure 3a,b show the normalized radial and hoop stresses, respectively, along the MN line.

It is not difficult to notice that the boundary conditions of the radial stress were satisfied at the hole periphery and at the limit radius. Furthermore, the peak value was progressively reduced as the stiffness ratio increased, and the prefixed radial stress at the limit radius was reached from the left with higher slopes. As far as the hoop stresses along the MN line are concerned, Figure 3b highlights two important aspects. The first one consists of the remarkable reduction in the maximum stress with respect to the homogeneous case. In fact, the hoop stresses associated with linear Young’s modulus distributions showed less stress concentration factors at the rim of the hole. To give some numerical values, the stress concentration factor decreased by approximately 39%, 58%, and 73% for the three considered stiffness ratios, respectively. The second aspect regards the stress uniformity along the MN line. In particular, the stress concentration factor decreased at the hole periphery, while a maximum value was recorded at some point along the radial direction. This inevitable stress rise is a consequence of the conservation of the integral of the hoop stress resulting from the equilibrium along the vertical MN line. Nevertheless, the rate of change of the maximum value of the hoop stress was considerably lower than the rate of change of the stress concentration factor at the rim of the hole, thus yielding lower variance values for the stress profiles along the MN line.

## 5. Discussion

**Figure 4:**
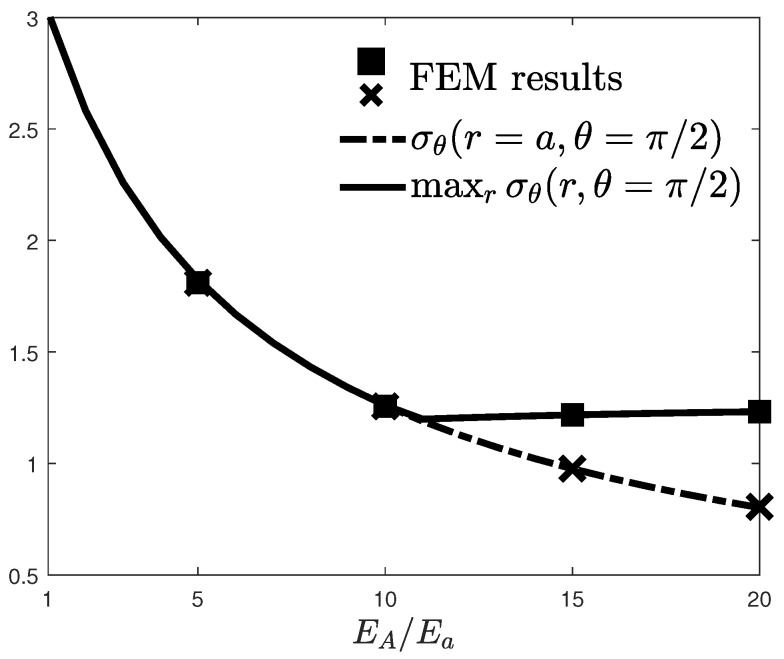
Hoop stress at the rim of the hole and its maximum value as functions of $E_{A}/E_{a}$. Scatter points refer to the numerical forecasts by means of the finite element method.

The above considerations shed the light on the existence of a particular value of the stiffness ratio, say $\bar{E_{A}/E_{a}}$, beyond which the maximum value for the hoop stress does not occur at the rim of the hole, but elsewhere (see Figure 4).

For the considered limit radius, such a value turned out to be 11. For halved and doubled limit radii, the $\bar{E_{A}/E_{a}}$ was 9 and 13, respectively, and the discrepancy between the corresponding peak stress values was less than 5%. Figure 4 shows both the hoop stress at the rim of the circular hole and its maximum value as functions of $E_{A}/E_{a}$. In agreement with Figure 3b, it is easy to show that both of these stresses were identical up to the aforementioned value, namely, for a stiffness ratio close to 11, after which the maximum value for the hoop stress seemed to increase very marginally.

For the sake of validation of these numerical results, a numerical model for the plate has been developed by means of the finite element methods (FEM). Due to symmetrical load and geometrical considerations, the numerical model consisted of the quarter of the plate and employed plane stress elements. Symmetric boundary conditions and the uniaxial load have been correctly applied to the model. The radial direction has been discretized into radial strips, each of which is isotropic and homogeneous and has the same mechanical properties. Adjacent layers present different properties such that the resulting piecewise constant variation mimics the linear Young’s modulus distribution. Maximum hoop stresses and the stress concentration factors have been computed and reported as scatter points in Figure 4, which shows a remarkable fit with the numerical results obtained by the method described in Section 3.

**Figure 5:**
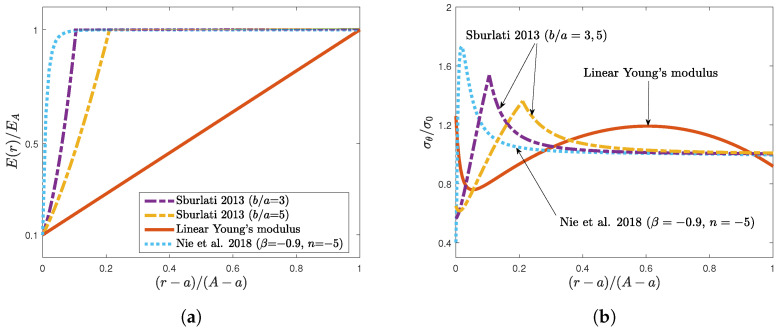
Comparison between the normalized hoop stresses along the MN line associated with a linear Young’s modulus (solid line), a generalized power law (dotted line), and when two functionally graded rings perfectly bonded to a homogeneous plate (dashed–dotted) were considered. (**a**) Young’s modulus distributions. (**b**) Associated hoop stresses along the MN line [17,18].

Eventually, some remarks on the worthiness of the considered Young’s modulus distribution will be addressed. This can be assessed by fixing an instance for the stiffness ratio, e.g., $E_{A}/E_{a}=10$, and comparing its elastic performance with that associated with different laws, which are largely employed in the literature (Figure 5a). Sound comparison criteria could include the maximum hoop stress along the radial domain and determing whether this value occurs at the hole periphery. For instance, for the specified inhomogeneity parameters β and *n* in the generalized power laws considered in [17], namely,

(15) $$E\left(r\right)=E_{A}\left[1+\beta {\left(\frac{r}{a}\right)}^{n}\right]\;,$$

the hoop stress at the rim of the hole was 40% of the applied load, while the peak stress was 73% more than the applied load (see the dotted line in Figure 5b). In [18], elastic stresses occurring in a functionally graded ring that was perfectly bonded to the circular hole were derived analytically when the stiffness in the ring was described by the power law,

(16) $$E\left(r\right)=E_{A}{\left(\frac{r}{b}\right)}^{m}\;,$$

where *b* is the radius of the ring (see the dahsed–dotted lines in Figure 5a). For fixed geometry and stiffness ratios, the grading factor *m* in Equation (16) can be computed as

(17) $$m=\frac{\log(E_{a}/E_{A})}{\log\left(a/b\right)}\;.$$

The hoop stress associated with two rings whose radii are three and five times the radius of the hole is shown in Figure 5b (dashed–dotted lines). Also here, in both cases, the maximum hoop stresses, whose values were, respectively, 54% and 36% more than the applied load, did not occur at the hole periphery. On the other hand, the maximum hoop stress for the linearly varying stiffness was 26% more than the applied load (solid line in Figure 5b), and this value decreased when the stiffness ratio assumed higher values (as shown in Figure 3b).

It is worth mentioning that most of the works available in the literature prefix the Young’s modulus distribution a priori and address, either analytically or numerically, the associated elastic problem. For instance, the solutions in the present work, as well as in [17,18], were obtained by heuristically choosing the parameters describing the stiffness throughout the plate. In fact, a proper choice of the parameters β and *n* in (15) and b/a and *m* in (16) led to enhanced values for the stress concentration factor, while in the present work, it was shown that there exists a slope *e* of the stiffness distribution that yields comparable results. However, we believe that a more intriguing approach should consist in finding the Young’s modulus distribution without prefixing its functional form beforehand. This approach could be introduced within an optimization problem aimed at reducing the stress concentration factor as much as possible. After discretizing the domain into a finite number of, e.g., radials strips, possible decision variables could include the Young’s modulus in each strip. Hence, the results of the present study can be useful while developing numerical codes, as these typically employ linear initial guesses before seeking the optimum solution.

## 6. Conclusions

The stress behavior of functionally graded plates with a circular hole and that are subject to a far-field uniaxial traction load has been addressed in this article. The Young’s modulus was allowed to vary linearly along the radial domain, while the Poisson’s coefficient was kept constant. A set of coupled linear differential equations describing the displacement, strain, and stress fields was analytically derived and numerically tackled for a few linear stiffness distributions that were associated with different slopes. Numerical solutions have been shown in graphical forms and discussed. Specifically, along the vertical MN line, the following concluding remarks are highlighted:

- Similar to the homogeneous case, a linearly varying stiffness distribution along the radial domain makes each radial strip become shortened and stretched along the radial and circumferential direction, respectively. These strain measures decrease in absolute value as the stiffness ratio increases.
- The peak value for the radial stress progressively decreases as the stiffness ratio increases.
- The stress concentration factor remarkably decreases as the stiffness ratio increases, thus confirming one of the most established findings in the literature.
- There exists a stiffness value beyond which the maximum hoop stress does not occur at the rim of the hole.

These findings have been validated by the finite element method and compared to the elastic performance associated with other stiffness distributions that have been commonly employed in the literature.

## Data Availability

No specific data was used.

## References

[1] Kirsch E.G. (1898). Die Theorie der Elastizität und die Bedürfnisse der Festigkeitslehre. Z. Des Vereines Dtsch. Ingenieure.

[2] Sevenois R.D.B., Koussios S. (2014). Analytic Methods for Stress Analysis of Two-Dimensional Flat Anisotropic Plates with Notches: An Overview. Appl. Mech. Rev..

[3] Kumar S.A., Rajesh R., Pugazhendhi S. (2020). A review of stress concentration studies on fibre composite panels with holes/cutouts. Proc. Inst. Mech. Eng. Part L J. Mater. Des. Appl..

[4] Koord J., Stüven J.L., Petersen E., Völkerink O., Hühne C. (2020). Investigation of exact analytical solutions for circular notched composite laminates under tensile loading. Compos. Struct..

[5] Nguyen-Hoang M., Becker W. (2022). Open holes in composite laminates with finite dimensions: Structural assessment by analytical methods. Arch. Appl. Mech..

[6] Safaei B., Pezeshki Z., Kotrasova K., Kormanikova E. (2022). Analysis of stress concentration at the edge of hole in plates with different widths by using FEM. IOP Conf. Ser. Mater. Sci. Eng. Civ. Eng. Conf..

[7] Jędrysiak J. (2013). Modelling of dynamic behaviour of microstructured thin functionally graded plates. Thin-Walled Struct..

[8] Jędrysiak J. (2016). Tolerance modelling of free vibration frequencies of thin functionally graded plates with one-directional microstructure. Compos. Struct..

[9] Abdalla H.M.A., Casagrande D., Moro L. (2020). Thermo-mechanical analysis and optimization of functionally graded rotating disks. J. Strain Anal. Eng. Des..

[10] Madan R., Bhowmick S. (2022). Optimum FG Rotating Disk of Constant Mass: Lightweight and Economical Alternatives Based on Limit Angular Speed. Iran. J. Sci. Technol. Trans. Mech. Eng..

[11] Moleiro F., Madeira J., Carrera E., Reddy J.N. (2020). Design optimization of functionally graded plates under thermo-mechanical loadings to minimize stress, deformation and mass. Compos. Struct..

[12] Wang Z.W., Zhang Q., Xia L.Z., Wu J.T., Liu P.Q. (2015). Stress analysis and parameter optimization of an FGM pressure vessel subjected to thermo-mechanical loadings. Procedia Eng..

[13] Karamanli A., Wattanasakulpong N., Lezgy-Nazargah M., Vo T.P. (2023). Bending, buckling and free vibration behaviours of 2D functionally graded curved beams. Structures.

[14] Nouri A., Astaraki S. (2014). Optimization of Sound Transmission Loss through a Thin Functionally Graded Material Cylindrical Shell. Shock Vib..

[15] Kubair D.V., Bhanu-Chandar B. (2008). Stress concentration factor due to a circular hole in functionally graded panels under uniaxial tension. Int. J. Mech. Sci..

[16] Mohammadi M., Dryden J.R., Jiang L. (2011). Stress concentration around a hole in a radially inhomogeneous plate. Int. J. Solids Struct..

[17] Nie G.J., Zhong Z., Batra R.C. (2018). Material tailoring for reducing stress concentration factor at a circular hole in a functionally graded material (FGM) panel. Compos. Struct..

[18] Sburlati R. (2013). Stress concentration factor due to a functionally graded ring around a hole in an isotropic plate. Int. J. Solids Struct..

[19] Sburlati R., Atashipour S.R., Atashipour S.A. (2014). Reduction of the stress concentration factor in a homogeneous panel with hole by using a functionally graded layer. Compos. Eng..

[20] Ashrafi H., Asemi K., Shariyat M. (2013). A three-dimensional boundary element stress and bending analysis of transversely/longitudinally graded plates with circular cutouts under biaxial loading. Eur. J. Mech. A/Solids.

[21] You L.H., Wang J.X., Tang B.P. (2009). Deformations and stresses in annular disks made of functionally graded materials subjected to internal and/or external pressure. Meccanica.

[22] Madan R., Bhowmick S., Saha K. (2019). Limit angular speed of L-FGM rotating disk for both temperature dependent and temperature independent mechanical properties. Mater. Today Proc..

[23] Abdalla H.M.A., Casagrande D., De Bona F. (2020). A Dynamic Programming Setting for Functionally Graded Thick-Walled Cylinders. Materials.

[24] Abdalla H.M.A., Casagrande D. (2021). An Intrinsic Material Tailoring Approach for Functionally Graded Axisymmetric Hollow Bodies Under Plane Elasticity. J. Elast..

[25] Nikbakht S., Kamarian S., Shakeri M. (2019). A review on optimization of composite structures part II: Functionally graded materials. Compos. Struct..

[26] Timoshenko S.P., Goodier J.N. (1970). Theory of Elasticity.

[27] Andreev V.I., Cybin N.Y. (2014). The Inhomogeneous Plate with a Hole: Kirsch’s Problem. Procedia Eng..

[28] Barber J.R. (2010). Elasticity, Solid Mechanics and Its Applications.

[29] Shampine L.F., Kierzenka J. (2001). A BVP Solver based on residual control and the MATLAB PSE. ACM Trans. Math. Softw..

